# Selections and Abstracts

**Published:** 1913-12

**Authors:** 


### SELECTIONS AND ABSTRACTS

### PHYSICIANS AND MODERN DANCES.

During the present era of social unrest, it is but natural that the emotions of the populace should be blown hither and thither. It is natura lthat standards of conduct should suffer a severe disturbance. The present day dancing gives evidence of the emotional unrest, the seeking of new fields for excitement, the gratification of impulses which has been roused by the free discussion of themes related to physical life.

When the two-step sought for favor there was widespread opposition to its introduction and the praises of the waltz were sung. The new dances with their origin in the underworld have made a strong bid for popular approval. It is time to ask, "Have they come to stay." Prophecy is a difficult art and a most dangerous one. Viewing modern dancers with their suggestiveness, exaggerated movements and appeals to sexuality, it would appear that we have reached the acme of social riot. One hears the statement constantly recurring, "These dances can be danced properly. They are harmless if the participants but conduct themselves with dignity and decorum." The implications of these statements are that definite action must be taken to insure safety in dancing the tango, the turkey trot, and the various other dances of animal names and nature.

Is there not a medical argument against these dances which has not been fully appreciated by parents, ministers, reformers, physicians and chaperons? Dance neuroses are making their appearance. Sexual neurasthenias, pelvic congestions, prostatorrhea, priapism, congested prostates, and spermatorrhea represent some of the evidences of the physical and psychical inluences of the dances which at present have secured a hold upon the dancing public. The fact that men are rushing to the dance, that old men are hastening to cavort in the gliding mazes, is indicative of the stimulating and rejuvenating qualities of the alleged graceful Terpsechorean fads of the day. That a few persons may withstand the physical abuses resulting from the peculiar rhythm, the close contact and sinuous movements does not counterbalance the pernicious effects of these actions upon the multitude. In the words of Marcus Aurelius, "What is not good for the swarm is not good for the bee."

The introduction of dance halls in connections with saloons, the establishment of dancing areas in cafes and restaurants, attests the popularity of the free revelries of dancing. The combination of the dance with alcoholic stimulants, the setting of the scene in apepaling lights and attractive color, the bewitchery of syncopated music, the diaphanous feminine attire, are all concomitants tending to accentuate the physical nature of modern dancing and are indicative of the real nature of the lustful appeal to the participants in this modern sexual diversion.

Among the the fine arts, dancing requires no defense. Its beauty, grace and ecstatic movements are crowned with the glory of joyousness and exhilaration. When, however, such popular healthful amusements evidence degeneration and are prostituted for the development of sensual stimuli, the dance no longer should receive the support of the intelligent and moral parents who aim to safeguard their growing children. "The light fantastic toe" offers an opportunity for exercise, development, amusement, and recreation and has a field of desirable usefulness. If art must be debased, let it remain in the brothel and not be flaunted in the dancing academy, the reception hall, and the home. It is as unwise as it is socially malicious to create unnecessary stimuli that irritate and augment the sex characteristics of the dancing population. The modern sensual dance is opposed to the best interests of society and tends to a marked though insidious deterioration of the morality of the dancing public. Let physicians counsel their patients in the light of their undertaking and unhesitatingly speak forth publicly the truth concerning the demoralizing physical and psychical effects of the degenerate erotic dances.—Ed., Medical Review of Reviews.

---

### UNITED STATES CIVIL-SERVICE EXAMINATION. MEDICAL ASSISTANT (MALE), JANUARY 12, 1914.

The United States Civil Service Commission announces an open competitive examination for medical assistant, for men only. From the register of eligibles resulting from this examination certification will be made to fill a vacancy in this position in the Bureau of Chemistry, Department of Agriculture, Washington, D. C., at 1,800 a year, and vacancies as they may occur in positions requiring similar qualifications, unless it is found to be in the interest of the service to fill any vacancy by reinstatement, transfer, or promotion.

The duties of this position will be to study the claims and representations made in conjunction with proprietary remedies, look up medical literature, assist in preparing cases, etc., under the Food and Drugs Act. A knowledge of French and German is desirable.

Competitors will not be assembled for examination, but will be rated on the following subjects, which will have the relative weights indicated.

SUBJECTS.

| | Subjects | Weights. |
|---|---|---|
| 1. | General education and medical training | 35 |
| 2. | Practical or professional experience and fitness | 45 |
| 3. | Publications or thesis | 20 |
| | Total | 100 |

Graduation from a medical school of recognized standing and at least three years' subsequent experience in the practice of medicine, or two years' subsequent experience in either pharmacological investigations or the actual examination of drug products with reference to the claims made therefor by manufacturers, are prerequisites for consideration for this position.

If a thesis is submitted under subject 3 it must present the results of original investigational work on the part of the applicant in some phase of medicine or pharmacology.

Statements as to training, experience, and fitness are accepted subject to verification.

Applicants must have reached their twenty-fifth but not their forty-fifth birthday on the date of the examination.

Under an act of Congress applicants for this examination must have been actually domiciled in the State or Territory in which they reside for at least one year previous to the date of the examination.

This examination is open to all men who are citizens of the United States and who meet the requirements.

Persons who meet the requirements and desire this exami ination should at once apply for application Form 304, and special form, to the United States Civil Service Commission, Washington, D. C.; the Secretary of the United States Civil Service Board, Post Office, Boston, Mass., Philadelphia, Pa., Atlanta, Ga., Cincinnati, Ohio, Chicago, Ill., St. Paul, Minn., Seattle, Wash., San Francisco, Cal.; Customhouse, New York, N. Y., New Orleans, La., Honolulu, Hawaii; Old Customhouse, St. Louis, Mo.; or to the Chairman of the Porto Rican Civil Service Commission, San Juan, P. R. No application will be accepted unless properly executed and filed, in complete form, with the Commission at Washington prior to the hour of closing business on January 12, 1914. In applying for this examination the exact title as given at the head of this announcement should be used.

Issued December 8, 1913.

---

### THE INJURED FINGER.

One of the most human, artistic and appealing pictures we have seen is "The Injured Finger," published by the Surgery Publishing Company of New York.

This depicts a representative type of the present day physician treating an injured finger of a street urchin, while his two pals are intently and sympathetically watching the operation.

We are sure that you would prize and your patients and friends would enjoy this beautiful reproduction, which is so much more natural, more appealing and more human and so different from most of the familiar office and library pictures depicting gruesome scenes.

Rome, Georgia, Dec. 16, 1913.

Editor Journal-Record of Medicine:

Will you kindly announce in your Journal that "A 60 days tour of the surgical clinics of Europe is being arranged for representative Southern surgeons under the auspices of the Georgia Surgeons' Club, to close with the meeting of the Congress of Surgeons of North America in London the latter part of July,

1914." It is the purpose to write each surgical master for a definite arrangement of clinics, which will be announced on the itinerary so that the details of the whole trip will be carefully worked before the tour begins and that will make a short tour valuable, an advantage that cannot be gained by an individual. As soon as Dr. R. Waters writes me I will furnish the progra mof clinics in N. O., Feb. 27 and 28, 1914. These two trips will not conflict as some will go to both and many will go to one who would not go to the other.

Yours truly,
R. M. Harbin, Secty-Treas.

---

### FRAUDULENT DRUGS.

A renewed crusade against fraudulent drugs is promised by Dr. Carl L. Alsberg, Chief of the National Bureau of Chemistry. We wish him godspeed and success, and so does every physician, druggist and sick man. There have been frequent complaints on the part of the manufacturers that unprincipled people are constantly making and selling imitations of standard drugs, or mixtures, and we are reluctantly compelled to believe the charges true. It is also charged that the old crime of short-weight tablets or pills is still practiced. There is no question that many soldiers and civilians perished of malaria during the Civil War because the quinine pills said to contain five grains of the drug, had but one grain or so. A few venturesome therapists began giving more, and published excellent results from 100 to 200 grains or more a day. Perhaps they were giving but 15 to 20 grains after all. We can well see that such fiendish practices as short-weighting may be fatal nowadays, and it is highly essential that packages should contain exactly what they claim. Misstatements are far more than mere frauds. Alsberg's greatest efforts will be directed against the most horrible of all frauds—the patent medicines which make impossible claims of efficiency with a view of extorting money from incurable people who are necessarily lacking in judgment and who will clutch at any straw. It seems that the former crusade against this unspeakable contemptible business has not been as successful as it should have been, because so many healthy people believe the advertised claims and insist upon buying the stuff for themselves or their friends and relatives. Demand creates supply as in every other "business." Manufacturers still supply amber beads to keep off "croup" as the American people demand the right to buy what they please. So it is a nice question as to how far paternalism can go without interfering with that personal liberty so necessary for safety in other affairs. The medical profession as a body has bitterly opposed every new and revolutionary measure of its pioneers, such as vaccination and the out door treatment of consumptives, so we cannot expect to be considered infallible guides. In spite of our opinion that a certain drug or mixture is useless or harmful, people will persist in using it, merely remarking that we have made mistakes before and may be making one now, and we richly deserve our present position. If we could have had the power we certainly would have forbidden the Pasteur method for curing rabies. It may be just as well, then, that the power to forbid the manufacture, advertisement and sale of patent medicines be surrounded by certain safe-guards, and that for the present we confine our efforts to prosecuting unquestionable frauds. There are enough of them, Heaven knows, to keep Alsberg busy the rest of his days, but we hope his days may be long in the land.

—Ed. American Medicine.

